# Sustainable Biochar and/or Melatonin Improve Salinity Tolerance in Borage Plants by Modulating Osmotic Adjustment, Antioxidants, and Ion Homeostasis

**DOI:** 10.3390/plants11060765

**Published:** 2022-03-13

**Authors:** Saad Farouk, Arwa Abdulkreem AL-Huqail

**Affiliations:** 1 Agricultural Botany Department, Faculty of Agriculture, Mansoura University, Mansoura 35516, Egypt; gadalla@mans.edu.eg; 2 Department of Biology, College of Science, Princess Nourah Bint Abdulrahman University, P.O. Box 84428, Riyadh 11671, Saudi Arabia

**Keywords:** biochar, eco-physiology, melatonin, osmotic adjustment, oxidative injury, salinity stress, water status

## Abstract

Salinity is persistently a decisive feature confining agricultural sustainability and food security in arid and semi-arid regions. Biochar (Bi) has been advocated as a means of lessening climate changes by sequestering carbon, concurrently supplying energy and rising crop productivity under normal or stressful conditions. Melatonin (Mt) has been shown to mediate numerous biochemical pathways and play important roles in mitigating multi-stress factors. However, their integrated roles in mitigating salt toxicity remain largely inexpressible. A completely randomized design was conducted to realize the remediation potential of Bi and/or Mt in attenuation salinity injury on borage plants by evaluating its effects on growth, water status, osmotic adjustment, antioxidant capacity, ions, and finally the yield. Salinity stress significantly decreased the plant growth and attributed yield when compared with non-salinized control plants. The depression effect of salinity on borage productivity was associated with the reduction in photosynthetic pigment and ascorbic acid (AsA) concentrations, potassium (K^+^) percentage, K^+^-translocation, and potassium/sodium ratio as well as catalase (CAT) activity. Additionally, borage plants’ water status was disrupted by salinity through decreasing water content (WC), relative water content (RWC), and water retention capacity (WTC), as well as water potential (Ψw), osmotic potential (Ψs), and turgor potential (Ψp). Moreover, salinity stress evoked oxidative bursts via hyper-accumulation of hydrogen peroxide (H_2_O_2_) and malondialdehyde (MDA), as well as protein carbonyl, which is associated with membrane dysfunction. The oxidative burst was connected with the hyper-accumulation of sodium (Na^+^) and chloride (Cl^−^) in plant tissues, coupled with osmolytes’ accumulation and accelerating plants’ osmotic adjustment (OA) capacity. The addition of Bi and/or Mt had a positive effect in mitigating salinity on borage plants by reducing Cl^−^, Na^+^, and Na^+^-translocation, and oxidative biomarkers as well as Ψw, Ψs, and Ψp. Moreover, Bi and/or Mt addition to salt-affected plants increased plant growth and yield by improving plant water status and OA capacity associated with the activation of antioxidant capacity and osmolytes accumulation as well as increased photosynthetic pigments, K^+^, and K^+^/Na^+^ ratio. Considering these observations, Bi and/or Mt can be used as a promising approach for enhancing the productivity of salt-affected borage plants due to their roles in sustaining water relations, rising solutes synthesis, progressing OA, improving redox homeostasis, and antioxidant aptitude.

## 1. Introduction

Borage (*Borago officinalis* L.; Boraginaceae), as a moderately salt-tolerant crop, represents an important medicinally cultivar that has about 20% γ-linolenic acid in the fixed oil [1]. It is commonly utilized for treating kidney disorders and diarrhea, impeding cholesterol formation, treating Alzheimer’s disease and gastrointestinal weakness, and decreasing diabetes spin-offs [2]. While borage is cultivated in many countries for medicinal uses, very few studies have been performed concerning salinity’s impact on its biochemical pathways and productivity.

Salinity represents the most critical eco-physiological constraints for agricultural productivity, which provokes desertification and limits crop production [3,4,5]. Salinization is increasing speedily and occupies virtually 20% of the global entire irrigated regions, and it is theorized to affect 50% of cultivated area in 2050, particularly in the majority of fertile regions worldwide [6]. The area of saline soil is expected to degenerate in various regions worldwide as a result of the application of salty water for irrigation, broken farming drainage structures, and universal warming [7]. Salinity causes considerable annual financial losses of 27.2 billion USD in irrigated agriculture [7]. Salinity adversely affects plant development [8], normal physio-biochemical processes [3,9], and nutrient uptake [5], and devastates photosystem II (PSII) reactions [10] plus contributes to molecular injury indirectly through the reactive oxygen species (ROS) [11]. The inequality among the assembly and eradication of ROS is able to perturb cellular homeostasis, which evokes oxidative anxiety, and accelerates biological macromolecules’ weakening and bio-membrane mutilation [10,12,13]. Neutralization of ROS injury is primarily done with superoxide dismutase (SOD), which dismutase superoxide anion to hydrogen peroxide (H_2_O_2_) and oxygen (O_2_) [14]. Afterward, H_2_O_2_ is packed up into H_2_O and O_2_ by catalase (CAT) and peroxidase (POD) [14]. In the interim, the antioxidant solutes contribute to adjusting the antioxidant enzyme activities and preserving typical metabolic processes, therefore raising plant stress tolerance [3,8,11]. Those so-called organic solutes decline water activity and decrease the cellular osmotic potential; thus, turgor and turgor-associated progressions possibly will be preserved in stress occurrences. Additionally, the organic solutes can proceed as osmoprotectants and stabilize subcellular bio-membranes [15]. As a typical reaction to stress features, plants acquire precise adaptive bio-molecular reactions, including osmotic adjustment (OA) [16]. Usually, OA is broadly recorded to be recognized as providing a high-energy reaction in maintaining cell turgor, desired for crop growth. The OA may be caused by the accumulation of numerous organic solutes and inorganic ions [17]. The accretion of free proline, soluble sugar, and protein in plant tissues can supply a sign of the level of stress tolerance provoked by osmoregulation [18].

As the main factor of the earth’s ecosystem, plant species can be classified as the main victim of salinity. Therefore, judgment-appropriate approaches to mitigate salinity injury should be given great precedence. Various endeavors have been completed to alleviate the dangerous effects of salinity, including the selection of salinity-tolerant cultivars; equally, profit has been tremendously constrained, as salt-tolerant genes are managed via numerous attributes, and their synchronized selection is not an easy assignment. Thus, using various plant activators like melatonin (Mt) and biochar (Bi) has achieved ground throughout the most recent decades as being a shot-gun approach [5,13].

Biochar (a charcoal-like substance) is formed from the thermochemical conversion of carbonaceous feedstock (pyrolysis or/and activation) in the lack or restricted occurrence of oxygen. These supplementations were presumably a result of mutually habitation activities and premeditated soil amendment by Amerindian populations earlier than the entrance of Europeans [19]. A huge quantity of Bi-derived C stocks stays in these soils at present, hundreds and thousands of years after they were abandoned. The total C storage is as elevated as 250 Mg C ha^−1^m^−1^ relative to representative rates of 100 Mg C ha^−1^m^−1^ in Amazonian soils derived from parallel parent material [20]. Such C storage in soils far surpasses the potential C sequestration in plant biomass even if bare soils were, hypothetically, restocked to chief forest containing about 110 Mg C ha^−1^ above ground [21]. Biochar as a soil conditioner can improve crop development by enhancing soil physio-biological attributes [21,22]. Most Bi investigations globally have been done on cultivated lands [19,23], yet there are only a few researchers examining the impacts of Bi under stressful conditions. These outcomes indicate that Bi can induce encouraging impacts via enhancing the physio-biochemical and biological trials of saline soils, including enrichment minerals nutrients and recover soil bulk density, stabilization of soil structure, accelerating water-holding capacity, and high cation exchange capability and microbial activity [24,25]. Furthermore, Bi may have the capacity to decrease salt injury following three mechanisms [22,26]: (1) transient binding of sodium (Na^+^) on its exchange position, and hence decreasing Na^+^ uptake; (2) rising potassium (K^+^) in soil solution, and so preserving Na^+^/K^+^ ionic balance to lessen Na^+^ uptake; and (3) rising soil moisture content, which may cause dilution effect and eventually causes a decline in Na^+^ uptake. Finally, a small number of researchers have illustrated the effect of Bi on oxidative anxiety and antioxidant capacity in salt-affected plants [27].

Melatonin represents the earliest phylogenetic indole substance found in entire biota [9,13,28], at 0.1 pg g^−1^ (FW) to 20~30 µg g^−1^ (FW) [29]. Since 1995, it was initially detected in vascular plants, and the assimilation processes and metabolic occupations have been largely recognized [28,30]. This examination twisted the awareness for Mt as a potential efficient feature to motivate stress tolerance. The Mt function in plants within a stressful environment is not completely implicit, and it introduces an enthusiastic calculation, whether it performs as an antioxidant and/or plant promoter [12,28,31,32]. The advantageous function of Mt in stress lessening is generally attributable to elevated photosynthesis, enhancement of redox homeostasis, mitigation of oxidative burst, and regulation of the stress-responsive gene expression implicated in signal transduction [9,11,30]. Evidence shows that Mt and its metabolites acquire both hydrophilic and hydrophobic properties; thus, it is considered as a powerful antioxidant in plant response to stress factors [30,32]. It can simply pass through bio-membranes and dispense to any aqueous section, i.e., cytosol, mitochondria, etc. Additionally, Mt not only frankly deactivates ROS and reactive nitrogen species (RNS), but also motivates antioxidant enzymes, thus motivating its antioxidant capacity that organizes the rupture of H_2_O_2_ in plants and defends them from oxidative anxiety [13,28,32,33]. Conversely, it is still indistinct whether such response of Mt against salinity is widespread for cultivars. Additionally, the mechanism of Mt-mediated salt tolerance is unclear. Consequently, it could be of enormous significance to exploit Mt as bio-activators for sustainable crop productivity without distressing the external environment.

Several types of research centered on the promotive effects of Mt and/or Bi on inducing salinity tolerance of several plants have been indicated previously. Whilst the individual encouraging impacts of Mt or Bi are well depicted, the synergistic impacts on salt-tolerance alleviation have hitherto to be elucidated. Consequently, the aims of the existing research were elucidating the regulatory approaches of Mt and Bi in mitigating salt-evoked injuries in terms of growth, yield, and some biochemical attributes that are modulated by salinity. We hypothesized that the individual and combined amendment of Mt and/or Bi possibly will lighten the oxidative injury by decreasing the Na^+^ uptake in borage. The outcome of the current investigation will possibly emphasize the importance of using Mt and/or Bi as regulatory agents for getting the greatest production from the salt-affected region in existing worldwide warming predictions.

## 2. Results

### 2.1. Organic Solutes and Water Relation

Organic solute was drastically amplified by either salinity or Bi amendment and/or Mt foliar application. The maximum value was recorded within Bi + Mt application under salinity that increased proline (133%) and soluble sugars (78%) relative to control plants. The data of leaf water potential (Ψw), osmotic potential (Ψs), turgor potential (Ψp), water content (WC), relative water content (RWC), water saturation deficit (WSD), and water retention capacity (WTC) established considerable variations in plant water status among control and salt stress in addition to Bi and/or Mt treatment (Table 1). Salinity considerably decreased the majority of plant water relation features, i.e., WC (22%), RWC (32%), Ψw (100%), Ψs (54%), Ψp (48%), and WTC (48%), and in the meantime enlarged WSD (133%) over control plants. The modifications stimulate OA status to be more positive for the water absorption. Application of Bi and/or Mt drastically improved RWC, WC, and WTC, whilst reducing WSD, Ψw, and Ψs, which induced OA aptitude and revival plant growth relative to unsprayed plants. The OA improved extremely with Bi and/or Mt application and may be predictable for maintaining Ψp of the leaf. Generally, the application of Bi+Mt treatment was more efficient than Bi or Mt application alone in rising leaf Ψp under a saline environment (Table 1).

### 2.2. Ion Accumulation and Translocation

The results presented in Table 2 indicated the alterations in K^+^, Na^+^, and Cl^−^ ion accumulation, and the K^+^/Na^+^ ratio as well as Na^+^ and K^+^ translocation (root to shoot) in the salt-stressed borage plants after Mt and/or Bi application. Under salinity, K^+^ and the subsequent K^+^/Na^+^ ratio were prominently (*p* < 0.05) reduced by 55% and 42% for K^+^ and 92% and 72% for the K^+^/Na^+^ ratio in either borage shoots and roots, respectively, above the non-salinized soil. Whereas Na^+^ and Cl^−^ were prominently increased in the shoots (799% and 399%) and in the roots (614% and 267%), respectively, when grown under salinity compared to control.

Nevertheless, Mt and/or Bi treatment remarkably enhanced K^+^ (136% and 45%) and K^+^/Na^+^ (390% and 218%) ratio in the shoot and root system accordingly, concerning the salinity devoid of Mt and/or Bi application. Moreover, Mt and/or Bi supply caused a considerable reduction in Na^+^ and Cl^−^ accretion in borage tissue compared to NaCl treatment (Table 2). The outcomes concerning K^+^ and Na^+^ translocation in borage tissue exposed to salinity with or without Mt and/or Bi supplementation are shown in Table 2. Borage plants exposed to salinity demonstrated that K^+^ translocation was reduced by 22% as compared to the control. Contrarily, salinity significantly (*p* < 0.05) enlarged Na^+^ translocation by 25% relative to the control plants. The addition of Mt+Bi under salinity enhanced K^+^ translocation (62%), while Na^+^ translocation was consequently greatly reduced by 38% relative to the salinized borage plants lacking Mt and/or Bi supply (Table 2).

### 2.3. Oxidative Biomarker

For deciding the function of Bi and/or Mt in the alleviation of salt-provoked oxidative damage, the oxidative biomarkers were estimated. In the current research, a substantial increase in H_2_O_2_ concentration was noticeable in salt-affected borage pants. On the other hand, Bi and/or Mt treatment allowed for a comparatively lower accrual of H_2_O_2_ in salt-stressed plants. The amendment of salt-stressed plants with Bi+Mt reduced H_2_O_2_ accumulation by 50% relative to salt-stressed plants alone (Table 3).

Malondialdehyde (MDA) can reflect the level of cellular injury. In this study, enhanced amounts of MDA (125%) were detected in salt-affected borage plants, relative to CK. However, the level of MDA concentration was significantly lesser in plants with Mt and/or Bi applied to the salt-stressed borage plants relative to non-treated salt-affected plants (Table 3). The most efficient in this regard was the soil supplementation with Bi associated with Mt spraying, which decreased MDA by 48% relative to the salt-affected plants (Table 3).

Proportionate to the control treatment, the protein carbonyl concentration under salinity increased drastically, by 72% (Table 3). When Bi and/or Mt were applied, the protein carbonyl concentration decreased. Application of Bi+Mt significantly reduced protein carbonyl concentration levels by 62%, corresponding to S plants, indicating that the Bi+Mt application had the inhibitor’s impact on ROS production of the borage plant.

Salinity caused an upsurge in the leakage of cellular electrolytes from 59.46% to 57.52% in the third upper leaves in the main stem of the borage plant compared to the control plants. Conversely, the application of Bi and/or Mt along with salinity significantly leveled off salt damage on the membrane permeability (%) by reducing the rate of cellular electrolytes compared to salt-treated plants (Table 3).

### 2.4. Antioxidant Enzymes

Under undesirable environments, plants utilize their antioxidant enzyme (i.e., SOD, POD, and CAT) to eradicate overload ROS for protecting plants from oxidative injury. The conducted study revealed a greater enhancement in the SOD (161%) and POD (16%) activity of salt-stressed plants than those of control plants. However, the extra addition of Bi and/or Mt in plant tissue of stressed borage plants further improved the activities of SOD and POD over salt-treated plants. The Bi+Mt had remarkably enhanced SOD and POD activity under salinity that was rose about 27% and 36%, respectively, compared to salt-affected plants (Table 4).

Salinity stress significantly reduced CAT activity by 53% relative to the control. Application of Bi and/or Mt in saline circumstances improved CAT activity above untreated salt-affected plants (Table 4). Amongst them, the raise in CAT activity was mainly noticeable under Bi+Mt treatment. The CAT activity level was 93% superior, correspondingly than the salt-affected plants, demonstrating that Bi+Mt most noticeably stimulated the CAT activity of borage plants (Table 4).

### 2.5. Antioxidant Solutes

Table 4 confirms that NaCl significantly raised phenols (51%) and flavonoids (Flav, 33%); however, it appreciably reduced AsA (53%) concentration in the borage plant comparative to control. Superior phenols, AsA, and Flav were recorded in Bi- and/or Mt-treated plants within the saline circumstances. This maintains that Bi and/or Mt have an extraordinary antioxidant activity via eliciting an antioxidant accumulation. The maximum phenols and Flav concentrations were acquired through Bi+Mt treatment under salinity. Phenols (98%) and Flav (100%) were drastically amplified in contrast to untreated control plants. Alternatively, the supreme AsA concentration was proofed with Bi+Mt supplementation, which increased by 49% compared to salinized plants.

### 2.6. Photosynthetic Pigment

Salinity induces an extraordinary lessening in total chlorophyll (35%) and carotenoid (73%) concentrations above the control plants. Application of Bi and/or Mt to salt-exposed plants prevents the salt-related photosynthetic pigment shortage via preserving a superior concentration compared with salt-affected plants. The Bi+Mt application provides supreme values of photosynthetic pigments compared with saline conditions, which increased it by 43% and 239%, respectively (Table 5).

### 2.7. Growth Parameters

The morphological traits of borage under salinity conditions, with or without Mt and/or Bi supplementation, are given in Table 5. Salinity repressed the growth trials of borage plants corresponding to control plants. Plant height, shoot fresh weight, shoot dry weight, and leaf area were significantly declined (*p* < 0.05) by 47%, 58%, 52%, and 69%, respectively, by salinity stress relative to the control plants (Table 5). Corresponding to salt-affected plants alone, the application of Mt and Bi individually or in combination reduces the inhibition of borage plant growth under salinity. The greatest values of plant height, shoot fresh weight, shoot dry weight, and leaf area of salt-affected borage plants were obtained by soil supplementation with Bi plus Mt application, compared to the saline medium alone, which increased it by 80%, 115%, 85%, and 150%, respectively (Table 5).

### 2.8. Yield Attributed

Influence of salinity and Bi and/or Mt on borage plant yield, i.e., seed yield/plant, seed index, oil percentage, and oil yield/plant, are delineated in Table 6. It proves that salinity considerably (*p* < 0.05) decreased borage yield corresponding to the control. Salinity decreased seed yield/plant, seed index, oil percentage, and oil yield/plant by 46%, 50%, 29%, and 61%, respectively, in relation to the control plants. Conversely, Bi and/or Mt drastically raised yield features relative to non-treated plants. Bi and/or Mt application under salinity diminished the NaCl injuries on yield attributes greater than their relevant untreated salt-affected plants. Compared with salt-affected plants, the greatest yield attributes were recorded under Bi+Mt treatment, which increased seed yield/plant, seed index, oil percentage, and oil yield/plant by 58%, 81%, 37%, and 118%, respectively (Table 6)

### 2.9. Fixed Oil Constituents

The GC study of borage seeds’ (Table 7) oil illustrated 11 well-known fatty. The oil was composed of 35.59 wt.% linoleic acid (18:2), 13.25 wt.% palmitic acid (16:0), 19.46 wt.% oleic acid (18:1), 3.58 wt.% stearic acid (18:0), 18.4 wt.% α-linolenic-ω6 acid (18:3n6), and 0.13 wt.% α-linolenic-ω3 acid (18:3n3), as shown in Table 7. Salinity modifying the fatty acid composition of borage oil increased stearic acid (4.03 wt.%), linoleic acid (35.85 wt.%), α-linolenic-ω6 acid (19.24 wt.%), and arachidic acid (0.25 wt.%). Additionally, the application of Bi+Mt under salinity considerably improved the quality of the oil by increasing palmitoleic acid (0.24 wt.%), stearic acid (4.01 wt.%), oleic acid (19.58 wt.%), linoleic acid (36.16 wt.%), α-linolenic-ω6 acid (19.52 wt.%), α-linolenic-ω3 acid (0.17 wt.%), and arachidic acid (0.27 wt.%).

## 3. Discussion

Salinity represents the major abiotic hazard that restricts plant growth and biomass production [5,8,10,28]. The overall decline in salt-affected plant growth possibly will be attributed to the impacts of salt on several metabolic pathways, ultrastructure modification, and molecular responses [4,9,11,34] associated with the current results. Salt stress can disrupt plant ion homeostasis, which causes ion poisoning, disturbs membrane stability, disturbs photosynthetic activity, decreases the cell water and osmotic potentials, and stimulates osmotic stress as well as extraordinarily lessening plant dry weight [5,9]. Additionally, salinity induces the interruption of chloroplasts and lessens cell water potential, which then shortly evokes stomatal closure and smaller CO_2_ incorporation, therefore withdrawing cell division [5,10]. Moreover, salinity evokes the accumulation of ROS, which accelerates oxidative injury and causes steady oxidative injury by the weakening of imperative cellular compounds, deactivates antioxidant capacity, interrupts plant–water relations, and lessens nutrient uptake [3,5,9,10,13].

Recently, Mt as an activator has attracted the attention of botanists [31,32,35]. For example, it provides biochemical and molecular resistance alongside numerous environmental stresses via its participation in indicating modifiable stress [9,13,28,32]. In the current investigation, Mt spraying improved plant growth of the borage plant, compatible with the outcomes of Ahmad et al. [13], who established that Mt lessens oxidative anxiety within a stress environment. Moreover, Mt decreased ethylene assembly throughout the regulation of the MaACO1 and MaACS1 expression [36]. Biochar (as organic fertilizer with activated carbon) amendment to saline soils is accounted for improving plant development. The encouraging impact of Bi on plant growth under regular or stressful conditions agrees with the results reported by Ibrahim et al. [27], Yang et al. [5], and El-Gamal et al. [37]. The direct growth improvement of Bi application was associated with increasing nutrients’ supply, i.e., calcium (Ca^2+^), magnesium (Mg), phosphorus (P), K^+^, and sulfur (S), etc., to the plant; however, the indirect method comprises enhancing soil physiochemical and biological features [5,22,23]. In addition, the impact of Bi stimulation is possibly accredited to a decrease in transpiration, preservation of a superior net photosynthetic rate (Pn), water use efficiency, RWC, the regulation of ribulose-bisphosphate carboxylase oxidase (RuBisco), and lastly, the amplification of the plant antioxidant capacity [5,27]. These obtained data recommended that the application of Mt and/or Bi drastically balances the attenuation in plants’ growth, and improves the stress tolerance by serving as ROS scavengers, enhancers of the nutrient uptake, and promising motivators of plants’ development under salinity.

Leaf yellowing is the key visible symbol of salinity injury in several plants [5,10,27]. The criticism of salinity on total chlorophyll and carotenoids are primarily coupled with a huge ROS assembly, which stimulates chloroplast clustering and destruction, as well as photosynthetic dysfunction [10,13]. The ROS hasten the structural alteration of pigment–protein complexes following three stages [38]: (i) the withdrawal and weakening of the antenna complex protein positioned in the grana; (ii) the substitute of Mg^2+^ with H^+^ ions, which evokes the pheophytinization of chlorophylls; and (iii) damage in the grana of the chloroplast. In this regard, Farouk and Arafa [34] found that salinity stress raised chlorophyll decomposition, possibly as a result of the buildup of Na^+^, which possibly hastens chloroplast ultrastructure modification. Moreover, stressful conditions accelerate the reduction in the content of the intermediaries Proto IX, Mg-Proto IX, and Pchlide throughout the chlorophyll assimilation [16], and reduce the hlD, ChlH, and Chl I-1 gene expression-encoding subunits of Mg-chelatase [39].

Comparable studies indicated that Mt [28,31,33,35] and/or Bi [5,23,40] supplementation raised total chlorophyll and carotenoids under normal or stressful environments, leading to a marinating greenish of the leaves. The impact of Bi and/or Mt on total chlorophyll and carotenoids contents is possibly associated with accelerating antioxidant enzyme activities or built up antioxidant capacity (Table 4), as confirmed by Ahmad et al. [13] and Chrustek and Olszewska-Stonino [41]. Generally, Mt represses salt inhibition of the ferredoxin gene PetFin rice [42], whereas ferredoxin defends chlorophyll from deprivation [43]. Additionally, Mt and/or Bi supplementation provokes Mg^2+^, carotenoid, and flavonoid buildup, which is attractive for chlorophyll assimilation or to guard chlorophyll against ROS. Lately, Mt spraying stimulated a visible reduction in the pheophorbide oxidase gene expression, senescence-correlated gene 12 (SAG12), and chlorophyllase gene expression [44].

Salinity episodic ion homeostasis induced a nutritional imbalance, leading to a buildup of poisonous ions, i.e., Na^+^ and Cl^−^, which is connected to the decreased K^+^ and K^+^/Na^+^ ratio values [3,8,9,28]. Under salinity, the foremost basis of declined K^+^ uptake may be caused by the ion imbalance and the aggressive uptake of Na^+^ and Cl^−^ with definite ions, i.e., potassium [4]. Moreover, the higher buildup of Na^+^ and Cl^−^ competed with the absorption of certain ions like K^+^ [12,28]. Additionally, extreme Na^+^ does not merely obstruct K^+^ absorption, but additionally upsets the cellular metabolic pathways, leading to nutritional confusions, restrictive absorption of vital ions, and eventually yield reduction [7].

Current studies have shown that Mt application improves plants’ ion homeostasis under salinity by increasing K^+^ and decreasing Na^+^, leading to a drastically higher K^+^/Na^+^ ratio. That was confirmed by Ren et al. [9], Wei et al. [32], and Zhang et al. [28]. Enhanced ion homeostasis is possibly connected to the upregulation of numerous genes, like the NHX1-encoding gene (responsible for the excessive transfer of Na^+^ into vacuolar; [45]), salt overly sensitive 1 (SOS1, responsible for conveying Na^+^ outside the cells; [46]), and AKT1-encoding gene (responsible for absorption of K^+^ from the soil and moving it into the roots; [47]). Thus, upregulating NHX1, SOS1, and AKT1 gene expression causes a rise in K^+^ and decrease Na^+^ in plant cells, thus enhancing the plant’s stress tolerance. Accordingly, NHX1 and SOS1 expression was superior in Mt-sprayed rapeseed seedlings to the non-treated ones that were linked with the lesser Na^+^/K^+^ ratio by decreasing Na^+^ and increasing K^+^ [48].

Bi supplementation significantly decreased Na^+^ entry into the cells, therefore enriching plant cells with K^+^, resulting in an increasing K^+^/Na^+^ ratio and improving plant growth under salinity [5,23]. Biochar has a high adsorption capacity, which possibly efficiently decreases Na^+^ uptake by plants [22,25]. Further, Bi serves as a direct supply of some ions, i.e., K^+^, Ca^2+^, and Mg^2+^ [22,25]. The elevated Ca^2+^ and Mg^2+^ liberated by Bi can move Na^+^ on the exchange site, additionally allowing for higher Na^+^ leakage and lesser soil salinity, thus improving soil properties [49].

In plants, a higher K^+^/Na^+^ ratio is the main feature, which assists salt-tolerant genotypes to grow healthy under salinity and to defend metabolic pathways by enhancing protein assimilation, regulating enzyme activities, and maintaining cell turgor [50]. The function of Bi and/or Mt in raising nutrient uptake is not completely implicit. In harmony with the current investigation, Ren et al. [9] confirmed that ion content was amplified in stress-affected plant tissue once Bi or Mt supplementation was administered, respectively. This promoting influence on neutralizing the corruption’s impacts of salinity on nutrient content may be associated with the enhancement in nutrient uptake, preserving membrane permeability, and/or probably enhancing root system progress [51].

Over-production of organic solutes (OS), i.e., proline and soluble sugars, in borage with salinity and Bi and/or Mt supplementation (Table 3) is an approach for protecting optimal cell Ψw and defensive nucleic acids and macromolecule structures [52]. According to the current outcomes and previous reports, salinity [3,9] or Bi supplementation [51] and Mt supplementation [9,13] elicited OS production in various plants, probably to supply superior protection alongside salt stress. Induction in OS accumulation has two possible physio-biochemical responses: (i) decreasing cell osmotic potential, and thus maintaining plant–water relations [16,17]; and (ii) stabilizing biomembranes and macromolecules by regulating salt stress-responsive gene expression [53]. Proline can work as an osmoprotectant, a protein preservative, and in control cell differentiation, which is vital for plant revival under stressful factors [54]. Proline may also perform as a prevailing ROS elimination and defend the cellular constituents alongside oxidative damage [12]. The higher accumulation of proline under salinity or Mt and/or Bi supplementation could be a result of the modification in the activities of proline assimilation, degrading enzymes, protein degradation, and/or gene (δ1-pyrroline-5-carboxylate synthetase, ProDH) expression [53]. Soluble sugars play a decisive role in the OA, turgidity, and bimolecular stability, and offer energy and carbon backbones, which assist cells to develop quickly and assimilate the requisite organic substances [55]. The superior production of soluble sugars under salinity or Mt and/or Bi application could be owed to increased RuBisco and carbonic anhydrase enzyme activities [53,56]. Incidentally, Mt spraying enhanced the production of soluble sugars by enhancing sucrose synthesis-related gene expression, which assists in preserving plants’ cellular integrity [57].

The preservation of elevated water status under stress is a prevailing outcome in crop cultivars. The WC, RWC %, and WTC levels in Bi and/or Mt application plants under saline conditions were retained at levels near to the control, as confirmed by previous research [28,32,51,58]. In the current investigation, salinity drastically decreased WC and RWC, but Bi- and/or Mt-treated borage plants established higher WC and RWC levels than untreated plants (Table 1), which delegated that Bi and/or Mt application played an important role in maintaining superior water relations and enhanced the salinity tolerance of borage. It is still undecided how Bi and Mt are able to maintain water status in plants within stressful environments. The increase in water preservation with Bi and/or Mt in stress-exposed plants might be coupled with (i) stomatal closure, (ii) a decrease in transpiration rate, or (iii) enhanced adventitious root growth. It is well known that Bi can maintain water in itself and accordingly elevate the water accessible to plants within stress conditions [58]. Additionally, Bi application enhanced soil aggregate stability, decreased soil bulk density, increased soil surface area, and increased soil water-holding capacity, resulting in enhancing soil moisture retention efficiently [24]. Additionally, Mt spraying might avoid crop water defeat through increasing leaf cuticle thickness [59].

Maintaining cell turgor next to decreasing Ψs is the main protection approach of plants within stressful conditions. Salinity and Bi and/or Mt supplementation hastened OS production, which probably decreased cell Ψs. Decreasing Ψw successively helps in preserving cell Ψp via an energetic declining of Ψs [16,28]. The reduction in RWC and Ψw is a decisive indication of stress confusion, which affects the water uptake into meristematic tissues [60]. The current data are in agreement with the previously accessible outcomes on the Ψw, Ψp, and RWC, representing decreased values with increasing stress times [28]. Biochar and/or Mt addition motivated leaf OS production as a result of decreased plant Ψw under stress, which is a recognized stress adaption to avoid plant dehydration and permanent wilting, and therefore preserve the finest turgor and regulate water uptake during transpiration [61]. The elevated hydrophilic inner surface area of the Bi particles, with enduring and storage holes, improves the water-holding capacity, and therefore decreases the soil evaporation rate [62]. Commonly, Bi and/or Mt enhanced the ion uptake, principally K^+^, which assists in preserving the plant Ψs [28] attributed to its extra cation concentration, which regulates the water status of the plant under a stressful environment. Kammann et al. [63] also documented a lessening in the transpiration rate in Bi-supplemented borage plants, which can be a cause for the enhanced Ψp and RWC of Bi-supplemented borage plants.

Under salinity, plants build up numerous methods for surviving, i.e., escape, tolerance, and avoidance, including OA capacity [64]. The OA represents the most applicable approach for tolerating environmental disorders [65]. Better performance of borage under salinity due to Bi and/or Mt application might be due to sustaining OA ability throughout harmonization with superior energetic solute accumulation and preservation of Ψw and Ψp, which is necessary for maintaining a typical development [65]. Accordingly, the cell Ψs is lessened, which successively sequentially preserves water homeostasis and enhances the aptitude of the cell to protect Ψp; this is desirable for enhancing water preservation, cell expansion, stomatal occupation, CO_2_ fixation, and stabilizing macromolecules, which induces the enhancement of photosynthesis competence and rising stress tolerance [65].

Mitochondria and chloroplasts’ oxidative pathways generate ROS during the plant’s life duration. Small ROS production illustrates the encouraging effect and acquires function as a signal through the cell-restoring process. Plant cells can preserve small ROS concentrations under normal circumstances, since the cell antioxidant capacity realizes equilibrium between the eradication and the assembly of ROS [66]. Correspondingly, under stressful conditions, these self-protective strategies are overdriven by the rapid temporary production of an enormous amount of ROS, identified as oxidative rupture [67]. As seen with earlier research, the exposure of borage plants to salinity results in an amplified buildup of H_2_O_2_, MDA, and protein carbonyl groups. It can be implicit that extreme ROS buildup potentially causes oxidative injury, which devastates the ROS mitigation system of plants, leading to membrane dysfunction through hyper-accumulation of MDA and protein oxidation [28,40,53].

Amongst ROS, extra awareness has been paid to H_2_O_2_, owing to its modest steadiness and capability to enter the cellular membrane as an unaffected substance in various plants. The existing data propose that oxi-reductive rupture is provoked by H_2_O_2_ buildup, which accelerates the over-production of MDA and cell membrane dysfunction, contributing to growth reduction. These judgments confirmed existing outcomes in previous research [13,28,41]. A commonly documented damage of salinity in plants is membrane dysfunction, the majority of which is probably through ROS gathering.

Consistently, most of the studies indicated that Mt and/or Bi alleviate ROS levels elicited by stress factors [13,28,32,33,51]. Constantly, the existing exploration assumed that Bi and/or Mt supplementation commonly moderated ROS injuries and preserved membrane permeability percentage; nevertheless, the maximum decline in oxidative biomarkers’ assembly was monitored in salt-affected plants that were treated with Bi+Mt. These outcomes suggest that Mt and/or Bi may mitigate salinity related to membrane damage by lessening H_2_O_2_ assembly (Table 3). On the contrary, Bi and/or Mt supplementation displayed less oxidative injury by hampering the creation of H_2_O_2_ and MDA (Table 3) [13,28,32,51]. However, this lessening is not only credited to enhance antioxidant enzymes, but can also be recognized as a non-enzymatic solute accumulation [11,33]. Even so, the function of Mt and/or Bi on the ROS neutralizing method was not precisely calculated. It can be confirmed that the decline in H_2_O_2_ assembly by Bi and/or Mt application is possibly due to H_2_O_2_ trapping via the extra-production of phenolics and the modulation of antioxidant enzyme activities (Table 5).

Melatonin performs as a universal antioxidant that interrelates with ROS and directly alleviates it, owing to its antioxidative efficiency and convenience [28,30,53]. Through the ROS-lessening cascade, a distinct Mt can scavenge about 10 ROS/RNS that vary from additional conservative antioxidants by upregulating the antioxidant enzymes’ coding gene expression and activation of mitochondrial uncoupling proteins (UCPs) [68]. Melatonin directly mitigates H_2_O_2_ and improves the antioxidant enzymes’ ability to eliminate H_2_O_2_ by upregulating the expression of Mt assimilation genes. This is possible since Mt, as an electron donor, directly reacts with ROS and their associated molecules, and Mt is itself oxidized to an indole cation free radical, which consecutively reacts with intracellular O_2_ and then oxidizes to constant N–acetyl N-formyl-5-methoxykinamine (5-MAFK). Under salinity, Mt spraying also drastically improved CAT, SOD, POD, and glutathione reductase relative to non-treated plants [28,32,33]. Furthermore, Mt interacts with ROS by enhancing antioxidant solutes (AsA-GSH) [69]. These results propose that exogenous Mt may perhaps progress cellular redox homeostasis by activating antioxidants’ capacity to nullify salt-induced ROS, consequently enhancing plant salt tolerance.

A little investigation into the impacts of Bi on oxidative anxiety and antioxidant enzyme activities in salt-affected plants was conducted. Accordingly, the supplementation of Bi drastically declined H_2_O_2_ and MDA concentrations under salinity (Table 3), demonstrating that Bi application efficiently declined oxidative anxiety. Comparable mitigations by Bi in oxidative injury and antioxidant enzyme activities of salt-affected plants were documented by Ibrahim et al. [27]. Torabian et al. [70] established that Bi decreased the assembly of ROS and DPPH activity in leaf cells and amplified the chlorophyll index under salinity. Additionally, the reduction of Na^+^ and Cl^−^ toxicity has an impact through adding the Bi to the soil and reducing O^2• −^ and H_2_O_2_, which accelerates a decline in antioxidant activities. These outcomes advocated that Bi could enhance plant biomass under salinity by decreasing oxidative anxiety and activating antioxidant enzymes.

Earlier research recognized that, within stressful conditions, crops proficiently moderated oxidative anxiety and maintained redox homeostasis via a serious ROS-mitigating system [13,51]. Normally, SOD is a decisive constituent of the plant’s antioxidant protection scheme that is utilized as the early stage of protection, which changes superoxide ions into H_2_O_2_ and oxygen. The POD is a prime enzyme that induces the fast elimination of H_2_O_2_ at its product sites with diverse organic and inorganic substances [71]. The boost in SOD and POD might have been a decisive reaction to the assembly of superoxide radicals by salt-provoked blocking of the mitochondria electron transport pathway [72]. Generally, CAT is the main common oxidoreductase responsible for breaking up H_2_O_2_ into H_2_O and O_2_, with a tremendously elevated turnover number (one CAT molecule could covert 6 million H_2_O_2_ molecules every second, thus combating salt-induced oxidative stress [67]). This reduction in CAT is certainly caused by the inhibitory effect of salt on the enzyme system itself, or probably through the activation of the enzyme-bound heme group.

The addition of Bi and/or Mt accelerates a speedy ROS eradication via improving SOD, POD, and CAT activities, which established that there was a proficient plant ROS-mitigating capacity (Table 4). This was confirmed in the experiment with borage, in that Mt and/or Bi supplementation scavenges H_2_O_2_ and induces the activation of the plant antioxidant system within salinity conditions [11,13,27,32,33]. Melatonin has an efficient antioxidant aptitude and enhances the activity of the antioxidant enzyme, therefore decreasing peroxidative injury [13,28,30,32]. This may have been attributable to upregulating the expression of genes (APX1/2, CAT1, and FSD1) transcripts associated with antioxidant enzymes and decreasing the deprivation of energetic substances to progress the activity of antioxidant enzymes and the expression of antioxidant genes [73]. Additionally, it controlled genes occupied in ascorbate assimilation, i.e., VTC4 and APX4, within salinity conditions. This may clarify the effect of Mt in improving the plant antioxidant aptitude [32]. Consequently, Bi and/or Mt application is able to reduce the salt-mediated destructive effect of the ROS scavenging system of borage plants and can enhance the resistance in plants under stress conditions.

Diverse non-enzymatic antioxidants occupy an adjacent function in plant protection mechanisms including AsA, soluble phenolic compounds, and Flav, which perform as ROS mitigators in coincidence with antioxidant enzymes or independently. Antioxidant substances are engaged in different metabolic pathways, and they not only have a crucial role in plant tolerance, but also proceed as enzyme cofactors, and additionally affect plant development [74]. The recent research assumed that the concentrations of AsA, phenols, and Flav were strictly decreased or increased, respectively, in the borage plants under stress. These remarks documented that NaCl aggravated oxidative injury in borage by deteriorating the antioxidant aptitude. In contrast, the supplementation of Bi and/or Mt improved the antioxidant control of borage by raising the level of AsA, phenols, and Flav, which led to decreasing the oxidative burden from improved salt tolerance.

Ascorbic acid is a chief plant antioxidant that occupies the main function in withdrawing ROS as a result of its ability to provide electrons in a wide range of enzymatic and non-enzymatic reactions. It is able to lessen H_2_O_2_ to H_2_O and O_2_ through the APX reaction [75]. Currently, the AsA concentrations of borage plants decreased significantly under salt stress. The decline in AsA level under salinity designated that a little AsA was oxidized to dehydroascorbic acid (DHA). After the application of Bi and/or Mt, the AsA concentration of every treatment was considerably enlarged (Table 4). The successful increase in AsA concentration by Bi and/or Mt is a tremendous sign of a decline in salinity-induced ROS assembly in salt-affected borage plants and lessens oxidative injury provoked by salinity [28]. This may be accelerated by Mt raising monoascorbate reductase (MDHAR), DHAR, and GR activity and hastening the restoration of AsA, thus ensuring moderately higher levels of AsA in borage plants within salinity and improving resistance to salinity.

Phenolic substances correlated straight with antioxidant success in plants, as they have electron-donating intermediaries, and consequently diminish extra ROS constructions [76]. Regularly, various outcomes that are similar to the assembly of phenol in plant tolerance are augmented within salinity [4,28], Mt [28], or Bi [37]. This accretion was probably provoked by activating the phenyl-propanoid pathways, thus enhancing the expression of the phenyl-aminolyase gene [77].

Flavonoids are broadly planted secondary products that are linked to their interface with the environment, including the ROS modulation status. Numerous investigations showed a boost in Flav in salt-stressed plants treated with or without being treated by Mt [28,78]. The biological distinctiveness of Flav is coupled with their prospective cytotoxicity and ROS eliminators. Flavonoids represent prevailing antioxidants achieved in the chelation of transition metals (Fe^2+^), which restrict ROS formation via the Fenton reaction [79].

The annual global financial beating induced by salinity is 27.5 billion USD [7]. The decline in crop yield under salinity was indicated earlier by Farouk and Arafa [34], Sofy et al. [4], and Yang et al. [5] and confirms the observations of the present study. For several salt-affected plant cultivars, the reduction in yield is regularly coupled with lesser photosynthesis levels [5,10]. There are two achievable causes for the salt-accelerated photosynthesis reduction: stomatal closure and chloroplast modification [5]. The parameters of chlorophyll fluorescence consist of maximum photochemical efficiency of PSII (Fv/Fm), photochemical quenching (qP), non-photochemical quenching [Y(NPQ)], and definite photochemical effectiveness of PSII [Y(II)], etc. [10]. Additionally, salinity stress decreased pollen viability and stigmatic receptivity [80].

The current study successfully established that Mt stimulated salt tolerance; which was validated by Yang et al. [5]. The encouraging impact of Mt on yield in diverse crops may be caused by their impacts on the rising carbonic anhydrase activity and accelerating RuBisco, which stimulated CO_2_ assembly and photosynthetic aptitude, which is reproduced by exploiting plant biomass production [28]. Furthermore, Mt acts to enhance vital functions in plant reproduction, i.e., floral differentiation, fertilization, and seed development, improves pollen –stigma interaction and improves stigmatic exudation and accelerates pollen tube development, which is desired for fertilization success [81,82].

Biochar applications are known to increase agricultural productivity [23,58], although the precise mechanistic influences of Bi have not been identified. Huang et al. [83] demonstrated that the rice yield was enlarged by 8%–10% after Bi application. There are numerous causes to explain why Bi may be able to boost the crop yield. Biochar supplementation stimulated leaf photosynthetic performance and diminished oxidative anxiety, leading to elevated plant biomass and yield under a saline environment [5]. Biochar is recognized to alter soil physiochemical characteristics and quality [25,84], thus distressing root biomass, optimizing root morpho-biochemical attributes, and subsequently boosting crop productivity [85]. Raising the soil pH can influence the ionic charge equilibrium within and outside the root [24], and this modification in the cation/anion ratio might contribute to pH alteration in the plant xylem sap [86]. Improved xylem sap pH may cause a considerable strengthening of ABA signaling causing stomatal closure in plants under salinity [87].

The oil was rich in polyunsaturated fatty acids, chiefly oleic and linoleic and linolenic acids, which have therapeutic importance and, additionally, the oil contained a little quantity of C18:3 linolenic-ω3 that may cause a superior oil stability to marketable oils [88]. For example, soyabean oil has up to 6.8% of linolenic-ω3, and it is less steady because of rapid oxidation compared to *Salicornia bigelovii* seed oil, which has merely 1.4% of this fatty acid [88]. The occurrence of saturated acids (17.17%, 16.67%, 16.03%) in borage seed oil in control, salt, and Bi+Mt under salinity stress, respectively, represent a chief quantitative variation against marketable oils. Intermediary principles of saturated acids were established in seeds from *Salicornia brachiata* (16.5%) [89] and the chenopod *Suaeda fruticosa* (17.0%) [90]. similarly elevated levels of palmitic acid (21.8%–29.4%) were established in seeds of salt fat and coastal dune halophytes (*Arthrocnemum macrostachyum*, *Haloxylon stocksii*, *Alhagi maurorum*, *Cressa cretica*, and *Halopyrum mucronatum*) from Asia [90].

## 4. Materials and Methods

### 4.1. Experimental Layout

A pot experiment was done under greenhouse at Mansoura city, Egypt (latitude 31°02′40.6″ N, longitude 31°22′40.3″ E, altitude 15 m above sea level) in the 2017/2018 season. A plastic pot (40 × 30 cm) was filled with 15 kg of experimental soil (clay loam texture with pH 7.30; total nitrogen 199 mg kg^−1^; available phosphorus 4.9 mg kg^−1^; available potassium 185 mg kg^−1^; organic substance 1.12%; dry bulk density 1.40 g cm^−3^), according to Motsara and Roy [91], and amended with 5 g of compound fertilizer (N:P_2_O_5_:K_2_O, 20:20:20). Ten healthy and uniform borage seeds (obtained from Medicinal and Aromatic Plant Research Dept., HRI, ARC, Giza, Egypt) were sown into each pot on 1 October 2017. The pots were set in a completely randomized design, which involved five treatments with five replicates: (1) non-saline soil devoid of NaCl or Bi or Mt, (2) saline soil with 5000 mg NaCl/kg soil, (3) saline soil with Bi at 5% air-dried soil, (4) saline soil with Mt spraying (100 µM), and (5) saline soil with Bi and Mt. The preferred salinity level was derived from a pilot testing, which utilized 1250, 2500, 5000, and 7500 mg NaCl/kg soil, for 4 days, and showed that plant wilting occurred under 7500 mg NaCl/kg soil; however, there was no noticeable wilting in plants under 1250, 2500, and 5000 mg NaCl/kg soil. Salinized soil was prepared by pouring the proper quantity of NaCl as designated before in every pot via irrigation water. Additionally, the appropriate Mt or Bi levels were selected based on our former investigation and further references [37,38,48]. Bi was added throughout preparing the pots at the desired concentration, whereas Mt foliar application (200 mL pot^−1^) was done thrice at 40, 50, and 60 days from cultivation, until dripping using a hand sprayer, whilst concurrently covering the pot surface to avoid the Mt solution from coming in reaching to the soil surface.

### 4.2. Sampling Date

The plants from all treatments were harvested at 90 days from sowing for evaluating morphological and biochemical attributes. All determinations were performed using five replicates except oil constituents, which were performed on three replicates only.

### 4.3. Morphological Characteristics

The plants were collected and we measured the plant height and shoot fresh weight; after that, samples were oven-dried at 70 °C for shoot dry weight assessment. In addition, the leaf area was measured according to the method of Koller [92].

### 4.4. Total Chlorophyll and Carotenoid Concentration

Total chlorophyll and carotenoid concentrations (mg g^−1^ FW) within the fifth upper leaf were extracted with ice-cold methanol supplemented with sodium carbonate and quantified spectrophotometrically (T60 UV-Visible spectrophotometer, PG Instrument Limits, UK), at 470, 653, and 666 nm following the Lichtenthaler and Welburn [93] method.

### 4.5. Ion Percentage

Sodium (Na^+^) and potassium (K^+^) were extracted by the Motsara and Roy [91] protocol and assessed by a flame photometer. On the other hand, chloride (Cl^−^, mg g^−1^ DW) was determined by 0.01 N of silver nitrate solution with 5% potassium dichromate as an indicator [94]. Additionally, K^+^ and Na^+^ translocation (from root to shoot) was designed following Malik et al.’s [95] equation.

(1) $$\mathrm{Translocation}\;\mathrm{factor}=\frac{\mathrm{Ionic}\;\mathrm{concentration}\;\mathrm{in}\;\mathrm{leaves}}{\mathrm{ionic}\;\mathrm{concentration}\;\mathrm{in}\;\mathrm{roots}}$$

### 4.6. Organic Solutes and Water Relations

Proline (mg g^−1^ FW) was estimated by a ninhydrin colorimetric scheme [96]. The shoot sample was homogenized in aqueous sulpho-salicylic acid (3%) and centrifugation. Therefore, the 2 mL reaction mixture was reacted with 2 mL of glacial acetic acid and 2 mL of acid ninhydrin in a test tube for 30 min at 100 °C in a water bath, and finally, toluene was added. The toluene part absorbance was read at 520 nm. Meanwhile, total soluble sugar concentrations (mg/g DW) were determined based on the anthrone colorimetric technique recorded by Sadasivam and Manickam [97].

Plant water relation attributes were quantified in the shoot tips. Shoot tips were weighted for achieving fresh weight (FW) and then floated in distilled water in a closed Petri dish for 8 h, to measure the turgid weight (TW). Finally, the plant samples were consequently dehydrated at 80 °C in a pre-heated oven for the dry weight (DW) assessment [98]. Relative water content (RWC) and water content (WC) were determined by the following equations.

WC (%) = (FW − DW) × 100/FW(2)

(3) $$\mathrm{RWC}\;(\%)=\frac{\mathrm{FW}\;-\;\mathrm{DW}}{\mathrm{TW}\;-\;\mathrm{DW}}\times 100$$

Additionally, plant water saturation deficit (WSD) and water retention capacity (WTC) were calculated via the Baque et al. [99] equations: WSD (%) = 100 − RWC, and WTC = TW/DW. 

Leaf water potential (Ψw) was judged next to the Chardakoves technique as explained by Taiz and Zeiger [100]. We defined the osmotic potential (Ψs) following the Van’t Hoff equation, Ψs (MPa) = −miRT, where m is the molality (moles per 1000 g), i is the ionization constant, R is the gas constant, and T is the temperature (k). Turgor potential (Ψp) was calculated as the differences between Ψw and Ψs [101]. Alternatively, osmotic adjustment (OA) was calculated as the difference in Ψ0 between the treated plants and the control ones [102].

### 4.7. Oxidative Biomarkers

Hydrogen peroxide (μM/g FW) was determined as per the Tariq et al.’s [103] protocol, by homogenizing the fresh plant tissue in chilled acetone, followed by centrifugation. The supernatant was treated with 0.1% titanium reagent and then with ammonium solution (25%); afterwards, centrifugation was done, and the yellow mixture optical density (OD) was read at 415 nm. The concentration of H_2_O_2_ was designed via a pure H_2_O_2_ standard curve, involving the extinction coefficient of H_2_O_2_ (μM^−1^cm^−1^).

Lipid peroxidation (nM MDA/g FW) was estimated by assessing malondialdehyde (MDA) production via the thiobarbituric acid (TBA) technique [104]. The MDA was extracted from 0.5 g leaf FW, with 0.1% trichloroacetic acid (TCA). The homogenate was centrifuged for 15 min at 10,000× *g*. Subsequently, 4 mL of 20% (*w*/*v*) TCA containing 0.5% TBA was poured into every 1 mL of the supernatant. This mix was excited at 95 °C for 30 min, chilled speedily, and centrifuged for 15 min at 10,000× *g*. The absorbance was calculated at 532 and 600 nm, and molar absorptivity of 155 mM^−1^cm^−1^ was applied to calculate the MDA concentration.

Additionally, the protein carbonyl group (nM/g FW), as an eventual result of protein oxidation, was estimated via colorimetrically following Levine et al.’s [105] protocol. Plant homogenates (0.5 mL) were mixed with 0.5 mL of 2,4-dinitrophenylhydrazine solution in a tube and incubated at laboratory temperature for 2 h with an occasional vortex. Subsequently, 20% trichloroacetic acid (0.05 mL) was decanted followed by centrifugation. The supernatant was removed, and the pellets were washed 3 times with ethyl acetate solution (1 mL). The protein, free of unreacted reagent, was suspended in guanidine hydrochloride solution (0.6 mL). Carbonyl concentration was calculated from maximum absorbance (390 nm) using a molar extinction coefficient of 22,000 L/(mol·cm).

Cell membrane permeability (MP) was estimated following the technique of Tariq et al. [103] with slight adaptations. Plant segments were allowed to deposit in 10 mL of distilled water for 6 h and then preliminary electrical conductivity (EC1; Hanna Instruments, UK) was recorded. The samples were heated at 1000 C for 15 min and chilled to laboratory temperature, and subsequently, conductivity was calculated once more (EC2). The MP% was established following this equation: MP% = (EC1/EC2) × 100.

### 4.8. Antioxidant Enzyme Assay

Antioxidant enzyme activity (unit mg^−1^ protein) was estimated in the main shoot tips (with two leaves). Shoot tips (0.5 g) were triturated in sodium phosphate buffer (50 mM, pH 7.0) with 1% soluble polyvinylpyrrolidine, and then centrifuged at 20,000× *g* for 15 min. Finally, the supernatant was utilized for the evaluation of the antioxidant enzyme activity.

Superoxide dismutase (EC 1.15.1.1) was achieved using the reaction mixture (3 mL) containing K-phosphate (150 mM, pH 7.8), methionine (13 mM), p-nitrobluetetrazolium chloride (NBT, 75 μM), riboflavin (2 μM), and EDTA (0.1 mM), which were evaluated with an illumination below 500 µmolm^−2^ s^−1^ PPFD [106]. One unit of SOD activity defined as the quantity of enzyme that causes 50% inhibition of NBT reduction below the assessment circumstance.

Peroxidase (EC 1.11.1.7) was determined as guiacol-stimulated peroxidation of H_2_O_2_ [85] with a few modifications. Phosphate buffer (50 mM) was utilized for the extraction of POD followed by centrifuging. The assay mixture was composed of phosphate buffer, H_2_O_2_, guaiacol, and enzyme extract. The OD at 436 nm was measured, and the molar extinction coefficient of 26.6 mM^−1^cm^−1^ was achieved for the enzyme activity measurement (1 unit = 1 µM H_2_O_2_ decline min^−1^).

Catalase (EC 1.11.1.6) was anticipated by assessing the prime speed of H_2_O_2_ defeat depending on the modified procedure of Chrysargyris et al. [106]. About 100 µL of enzyme extract was poured into 50 mM K phosphate buffer (pH 7.0) and 10 mM H_2_O_2_ for a total amount of 3 mL. Enzyme activity was considered at 240 nm for 2 min. The extinction coefficient of 39.4 mM^−1^cm^−1^ was exploited to calculate CAT activity (1 unit = 1 mM of H_2_O_2_ decline min^−1^).

### 4.9. Antioxidant Metabolites

The scheme utilizing 2.6-dichlorophenol indophenol and Folin–Ciocalteu techniques was occupied for assessing the levels of ascorbic acid (AsA, mg/g FW) and total phenol concentration (mg gallic/g DW) in the shoot tips, respectively [97].

The flavonoids’ concentration (Flav, mg quercetin g^−1^ FW) was calculated by the aluminum chloride (AlCl_3_) procedure according to Sevket et al. [107]. A 1.0 mL portion of plant extract was placed in a 10 mL volumetric flask, distilled water (4 mL) was poured, 0.3 mL of sodium nitrite (NaNO_2_) was added, and the mixture was mixed carefully. After 5 min, 3 mL of AlCl_3_ was poured and the solution was mixed thoroughly for 5 min; then, we added 2 mL of sodium hydroxide, 2.4 mL of distilled water was added, and absorbance was calculated at 510 nm with quercetin utilized as the standard for calibration.

### 4.10. Yield and Oil Percentage

At harvesting, seed yield/plant, seed index (100 seed weight, g), oil percentage, and oil yield/plant were determined. The oven-dried powdered seeds were extracted for 6 h in a Soxhlet extractor with n-hexane as a solvent, and the oil-containing hexane was filtered by the Whatman No. 1 filter paper, and subsequently excited at 60 °C to get rid of the final traces of the solvent for about 2–3 h [108].

### 4.11. Fatty Acid Profiles

For the control, salinity, and Bi+Mt treatment under salinity treatment, the seed oil was collected for determination of its constituents. Fatty acid methyl esters (FAME) were propagated from lipid by using a quick technique along with the ISO 12966-2 protocol. FAME was produced by trans-esterification with methanolic potassium hydroxide as a transitional phase before saponification took place. Around 0.1 g of the oil was cited in a 5 mL screw-top test tube, and isooctane (2 mL) was poured into the tube and the tube was shaken. Methanolic potassium hydroxide solution (0.1 mL, 2 N) was set on the cap fixed with a PTEE joint, the cap was tightened and shaken dynamically for the 30 s, and the tube was left to stratify while awaiting the upper solution and then was poured. The isooctane solution is appropriate for injection into the gas-liquid chromatography (GLC). GLC equipped with a DB-23 column (60 m × 0.25 mm × 0.23 µm). The carrier gas was N_2_ with a flow speed of 1.5 mL/min and a splitting ratio of 1:50. The injector temperature was 250 °C and that of the flame ionization detector (FID) was 280 °C. The peaks were identified by comparing the retention times gained with standard methyl esters.

### 4.12. Statistical Analysis

Data were examined by one-way analysis of variance (ANOVA). Means were compared by the multiple mean comparison statistic and Tukey’s Honestly Significant Difference (HSD) test with COSTATC statistical package (CoHort software, 2006; North Carolina, USA). Statistical significance was set at *p* < 0.05. Data were presented as means ± standard error (SE) of five independent biological replications.

## 5. Conclusions

Exogenous application of Bi and/or Mt will potentially lighten the oxidative stress by improving the antioxidant power of plants as well as decreasing Na^+^ accumulation. Moreover, these treatments can lessen salt stress by encouraging the buildup of osmoprotectants, i.e., soluble sugars and proline. The outcome offers a speculative base for Bi and/or Mt to alleviate salt stress. Extra studies are necessary to understand the accurate functions of Bi and/or Mt under salinity. As Mt and/or Bi are secure for animals and humans and low priced, so a conditioning method utilizing them as plant activators is possibly a consistent, reasonable, and cost-effective means for producing borage plants in saline soil.

## Figures and Tables

**Table 1:** The effect of melatonin (Mt) and/or biochar (Bi) supply on osmolyte accumulation, plant water relations, water potential (mega-pascal, Mpa), osmotic potential (Mpa), turgor potential (Mpa), and osmotic adjustment (Mpa) of borage plants grown in a saline medium (S, 5000 ppm NaCl).

	Proline (mg/g FW)	Soluble Carbohydrates (mg/g DW)	WC	RWC	WSD	WTC	Water Potential	Osmotic Potential	Turgor Potential	Osmotic Adjustment
Control	4.625 ± 0.12 ^e^	145 ± 5.78 ^c^	86.87 ± 0.92 ^a^	80.31 ± 0.83 ^a^	19.68 ± 0.836 ^c^	9.33 ± 0.63 ^a^	−0.423 ± 0.012 ^a^	−0.626 ± 0.013 ^a^	0.203 ± 0.001 ^b^	0.000 ^e^
S	6.829 ± 0.10 ^d^	198 ± 6.10 ^b^	66.91 ± 2.38 ^c^	54.03 ± 1.43 ^c^	45.96 ± 1.433 ^a^	4.82 ± 0.48 ^c^	−0.850 ± 0.020 ^b^	−0.955 ± 0.022 ^b^	0.105 ± 0.002 ^e^	0.329 ± 0.019 ^d^
S+Mt	9.261 ± 0.23 ^b^	242 ± 6.48 ^ab^	72.24 ± 0.98 ^c^	73.71 ± 2.17 ^ab^	26.28 ± 2.179 ^bc^	4.54 ± 0.10 ^c^	−1.347 ± 0.020 ^d^	−1.514 ± 0.022 ^d^	0.167 ± 0.002 ^c^	0.887 ± 0.036 ^b^
S+Bi	8.176 ± 0.15 ^c^	200 ± 15.90 ^b^	79.17 ± 1.71 ^b^	71.37 ± 1.95 ^b^	28.62 ± 1.95 ^b^	6.39 ± 0.42 ^bc^	−1.030 ± 0.006 ^c^	−1.158 ± 0.007 ^c^	0.127 ± 0.001 ^d^	0.531 ± 0.020 ^c^
S+Mt+Bi	10.78 ± 0.05 ^a^	259 ± 11.71 ^a^	85.01 ± 0.19 ^ab^	78.62 ± 0.75 ^a^	21.37 ± 0.75 ^c^	8.22 ± 0.11 ^ab^	−1.757 ± 0.014 ^e^	−1.975 ± 0.016 ^e^	0.218 ± 0.001 ^a^	1.348 ± 0.015 ^a^
*p* value	***	***	***	***	***	***	***	***	***	***
LSD at 0.05	0.474	31.60	4.562	4.861	4.861	1.299	0.049	0.055	0.006	0.006

Values represent the means of five replicates, and the means not sharing the identical small letters in every column differ significantly from each other, consistent with the honest significant differences (HSD) test at a 5% probability altitude. Levels of significance are represented by *** *p* ≤ 0.001, and ns-not significant. (RWC, relative water content; WC, water content; WSD, water saturation deficit; WTC, water retention capacity).

**Table 2:** The effect of melatonin (Mt) and/or biochar (Bi) supply on some ions of borage plants grown in a saline medium (S, 5000 ppm NaCl).

	Shoot				Root				K^+^ Translocation	Na^+^ Translocation
	Potassium (K^+^)%	Sodium (Na^+^)%	K^+^/Na^+^ Ratio	Chloride (Cl^−^, mg/g DW)	K^+^%	Na^+^%	K^+/^Na^+^ Ratio	Cl^−^ (mg/g DW)		
Control	2.49 ± 0.11 ^a^	0.107 ± 0.00 ^e^	23.27 ± 1.07 ^a^	16.50 ± 0.94 ^e^	5.58 ± 0.12 ^a^	0.283 ± 0.10 ^d^	5.676 ± 1.05 ^a^	28.81 ± 1.89 ^d^	0.446 ± 0.03 ^b^	0.378 ± 0.03 ^b^
S	1.11 ± 0.05 ^c^	0.962 ± 0.00 ^a^	1.637 ± 0.06 ^d^	82.36 ± 4.98 ^a^	3.22 ± 0.16 ^b^	2.022 ± 0.06 ^a^	1.543 ± 0.50 ^de^	106 ± 2.45 ^a^	0.344 ± 0.01 ^c^	0.475 ± 0.06 ^a^
S+Mt	2.08 ± 0.04 ^b^	0.405 ± 0.00 ^c^	4.118 ± 0.13 ^c^	49.22 ± 0.47 ^b c^	3.88 ± 0.23 ^b^	1.147 ± 0.09 ^c^	3.382 ± 0.08 ^c^	67.21 ± 0.47 ^bc^	0.536 ± 0.05 ^a^	0.353 ± 0.02 ^b^
S+Bi	1.79 ± 0.02 ^bc^	0.697 ± 0.00 ^b^	3.156 ± 0.03 ^cd^	60.11 ± 2.87 ^b^	3.27 ± 0.28 ^b^	1.781 ± 0.03 ^b^	1.836 ± 0.10 ^d^	71.47 ± 0.47 ^b^	0.547 ± 0.02 ^ab^	0.391 ± 0.00 ^b^
S+Mt+Bi	2.62 ± 0.12 ^a^	0.326 ± 0.00 ^d^	8.030 ± 0.15 ^b^	38.34 ± 1.63 ^d^	4.68 ± 0.12 ^a^	1.113 ± 0.08 ^c^	4.916 ± 0.05 ^b^	59.64 ± 2.45 ^c^	0.559 ± 0.02 ^a^	0.292 ± 0.00 ^c^
*p* value	***	***	***	***	***	***	***	***	***	***
LSD at 0.05	0.266	0.017	1.543	8.56	0.626	0.249	1.656	5.659	0.102	0.109

Values represent the means of five replicates, and the means not sharing the identical small letters in every column differ significantly from each other, consistent with the honest significant differences (HSD) test at a 5% probability altitude. Levels of significance are represented by *** *p* ≤ 0.001, and ns-not significant.

**Table 3:** The effect of melatonin (Mt) and/or biochar (Bi) supply on oxidative biomarkers of borage plants grown in a saline medium (S, 5000 ppm NaCl).

	H_2_O_2_ (μM/g FW)	MDA (nM/g FW)	Protein Carbonyl (nM/g FW)	Membrane Permeability%
Control	18.75 ± 0.19 ^c^	9.59 ± 0.47 ^d^	19.66 ± 0.74 ^b^	59.46 ± 0.78 ^c^
S	51.05 ± 2.65 ^a^	21.60 ± 0.53 ^a^	33.97 ± 2.02 ^a^	87.52 ± 3.54 ^a^
S+Mt	26.78 ± 0.57 ^b^	14.82 ± 1.68 ^bc^	23.87 ± 1.44 ^b^	72.44 ± 1.29 ^b^
S+Bi	27.83 ± 1.50 ^b^	16.81 ± 0.94 ^b^	25.15 ± 1.29 ^b^	74.37 ± 0.90 ^b^
S+Mt+Bi	25.48 ± 2.15 ^bc^	11.19 ± 0.74 ^cd^	20.94 ± 0.49 ^b^	72.03 ± 1.86 ^b^
*p* value	***	***	***	***
LSD at 0.05	5.33	3.092	4.154	6.16

Values represent the means of five replicates, and the means not sharing the identical small letters in every column differ significantly from each other, consistent with the honest significant differences (HSD) test at a 5% probability altitude. Levels of significance are represented by *** *p* ≤ 0.001, and ns-not significant. (H_2_O_2_, hydrogen peroxide; MDA, malondialdehyde).

**Table 4:** The effect of melatonin (Mt) and/or biochar (Bi) supply on the antioxidant system of borage plants grown in a saline medium (S, 5000 ppm NaCl).

	Antioxidant Enzymes (unit/mg Protein)			Antioxidant Solutes		
	Superoxide Dismutase	Peroxidase	Catalase	Ascorbic Acid (mg/g FW)	Phenol (mg Gallic acid/g DW)	Flavonoid (mg quercetin/g FW)
Control	22.02 ± 1.38 ^c^	16.79 ± 1.25 ^b^	65.15 ± 0.47 ^a^	0.240 ± 0.008 ^a^	6.730 ± 0.15 ^c^	1.56 ± 0.07 ^d^
S	57.63 ± 3.39 ^b^	19.55 ± 0.60 ^b^	30.52 ± 0.65 ^e^	0.111 ± 0.004 ^d^	10.19 ± 0.70 ^b^	2.09 ± 0.01 ^c^
S+Mt	71.35 ± 4.81 ^ab^	26.20 ± 1.74 ^a^	51.02 ± 0.75 ^c^	0.182 ± 0.002 ^bc^	12.39 ± 0.26 ^ab^	2.89 ± 0.09 ^ab^
S+Bi	65.35 ± 2.49 ^ab^	25.89 ± 0.75 ^a^	44.22 ± 1.37 ^d^	0.174 ± 0.008 ^c^	10.70 ± 0.58 ^b^	2.58 ± 0.17 ^bc^
S+Mt+Bi	73.73 ±1.57 ^a^	26.61 ± 1.69 ^a^	59.11 ± 0.60 ^b^	0.222 ± 0.017 ^ab^	13.37 ± 0.44 ^a^	3.12 ± 0.11 ^a^
*p* value	***	***	***	***	***	***
LSD at 0.05	9.495	4.092	2.628	0.030	1.505	0.337

Values represent the means of five replicates, and the means not sharing the identical small letters in every column differ significantly from each other, consistent with the honest significant differences (HSD) test at a 5% probability altitude. Levels of significance are represented by *** *p* ≤ 0.001, and ns-not significant.

**Table 5:** The effect of melatonin (Mt) and/or biochar (Bi) supply on plant growth trials and photosynthetic pigment concentration of borage plants grown in a saline medium (S, 5000 ppm NaCl).

	Plant Height (cm)	Shoot FW (g)	Shoot DW (g)	Leaf Area (cm^2^)	Total Chlorophyll (mg/g FW)	Total Carotenoids (mg/g FW)
Control	81.1 ± 6.94 ^a^	1266 ± 52.6 ^a^	209.3 ± 7.76 ^a^	1136 ± 56.40 ^a^	1.85 ± 0.05 ^a^	0.786 ± 0.03 ^a^
S	42.6 ± 0.72 ^b^	531 ± 14.28 ^d^	99.75 ± 3.47 ^d^	351.2 ± 14.68 ^d^	1.20 ± 0.10 ^b^	0.206 ± 0.09 ^b^
S+Mt	66.5 ± 5.85 ^ab^	1039 ± 18.28 ^bc^	159.7 ± 3.48 ^bc^	734.3 ± 17.75 ^b^	1.60 ± 0.19 ^ab^	0.696 ± 0.04 ^a^
S+Bi	60.7 ± 6.57 ^ab^	914 ± 25.75 ^c^	140.9 ± 7.37 ^c^	572.6 ± 30.26 ^c^	1.55 ± 0.10 ^ab^	0.586 ± 0.02 ^a^
S+Mt+Bi	76.9 ± 3.66 ^a^	1146 ± 31.46 ^ab^	184.6 ± 6.13 ^ab^	879.4 ± 26.44 ^b^	1.72 ± 0.09 ^ab^	0.699 ± 0.02 ^a^
*p* value	**	***	***	***	*	***
LSD at 0.05	16.65	99.29	18.72	102.8	0.380	0.159

Values represent the means of five replicates, and the means not sharing the identical small letters in every column differ significantly from each other, consistent with the honest significant differences (HSD) test at a 5% probability altitude. Levels of significance are represented by * *p* ˂0.05, ** *p* ≤ 0.01; *** *p* ≤ 0.001, and ns-not significant. (FW, fresh weight; DW, dry weight).

**Table 6:** The effect of melatonin (Mt) and/or biochar (Bi) supply on seed and oil yield of borage plants grown in a saline medium (S, 5000 ppm NaCl).

	Seed Yield/Plant	Seed Index	Oil %	Oil Yield/Plant
Control	12.76 ± 0.22 ^a^	17.19 ± 0.27 ^a^	33.18 ± 0.39 ^a^	4.23 ± 0.12 ^a^
S	6.890 ± 0.17 ^d^	8.466 ± 0.36 ^e^	23.43 ± 0.39 ^d^	1.61 ± 0.06 ^d^
S+Mt	9.960 ± 0.37 ^b^	14.12 ± 0.16 ^c^	30.49 ± 0.34 ^bc^	3.03 ± 0.14 ^bc^
S+Bi	8.733 ± 0.26 ^c^	12.91 ± 0.13 ^d^	29.27 ± 0.43 ^c^	2.55 ± 0.11 ^c^
S+Mt+Bi	10.93 ± 0.21 ^b^	15.38 ± 0.27 ^b^	32.22 ± 0.34 ^ab^	3.52 ± 0.10 ^b^
*p* value	***	***	***	***
LSD at 0.05	0.822	0.811	1.206	0.368

Values represent the means of five replicates, and the means not sharing the identical small letters in every column differ significantly from each other, consistent with the honest significant differences (HSD) test at a 5% probability altitude. Levels of significance are represented by *** *p* ≤ 0.001, and ns-not significant.

**Table 7:** GLC fractions of borage oil of selected treatment.

Fatty Acids	Control	Salinity (5000 ppm NaCl)	Salinity + Bi (5%) + Mt (100 µM)	*p* Value	LSD at 0.05
Myristic acid (C14:0)	0.08 ± 0.001 ^b^	0.08 ± 0.001 ^b^	0.09 ± 0.001 ^a^	*	0.001
Palmitic acid (C16:0)	13.25 ± 0.005 ^a^	12.15 ± 0.004 ^b^	11.61 ± 0.007 ^c^	**	0.841
Palmitoleic acid (C16:1)	0.21 ± 0.009 ^b^	0.19 ± 0.004 ^c^	0.24 ± 0.007 ^a^	**	0.752
Margaric acid (C17:0)	0.05 ± 0.000	0.06 ± 0.000	0.05 ± 0.000	ns	
Stearic acid (C18:0)	3.58 ± 0.081 ^b^	4.03 ± 0.087 ^a^	4.01 ± 0.072 ^ab^	*	0.004
Oleic acid (C18:1)	19.46 ± 0.001	19.13 ± 0.001	19.58 ± 0.001	ns	
Linoleic acid (C18:2)	35.59 ± 0.004 ^b^	35.85 ± 0.004 ^b^	36.16 ± 0.002 ^a^	*	0.017
α-Linolenic acid (Omega-6, C18:3n6)	18.14 ± 0.001 ^ab^	19.24 ± 0.002 ^a^	19.52 ± 0.003 ^a^	*	0.020
α-Linolenic acid (Omega-3, C18:3n3)	0.13 ± 0.00 ^b^	0.13 ± 0.001 ^b^	0.17 ± 0.002 ^a^	**	0.006
Archidic acid (C20:0)	0.21 ± 0.004 ^c^	0.25 ± 0.003 ^b^	0.27 ± 0.004 ^a^	**	0.018
Cis-11-Eicosenoic acid (C20:1)	3.02 ± 0.001 ^b^	3.79 ± 0.004 ^a^	3.98 ± 0.007 ^a^	*	0.009
Total saturated%	17.17 ± 0.007 ^a^	16.57 ± 0.006 ^ab^	16.03 ± 0.002 ^b^	*	0.098
Total unsaturated%	76.55 ± 0.006 ^b^	78.33 ± 0.006 ^b^	79.65 ± 0.005 ^a^	**	1.001

Values represent the means of three replicates, and the means not sharing the identical small letters in every row differ significantly from each other, consistent with the honest significant differences (HSD) test at a 5% probability altitude. Levels of significance are represented by * *p* ˂0.05, ** *p* ≤ 0.01, and ns-not significant.

## Data Availability

Not applicable.

## References

[1] Gilani A.H., Bashir S., Khan A.-U. (2007). Pharmacological basis for the use of *Borago officinalis* in gastrointestinal, respiratory and cardiovascular disorders. J. Ethnopharmacol..

[2] Tewari D., Bawari S., Patni P., Sah A.N., Nabavi S.M., Silva A.S. (2019). Borage (*Borago officinalis* L.). Nonvitamin and Nonmineral Nutritional Supplements.

[3] Farouk S., Elhindi K.M., Alotaibi M.A. (2020). Silicon supplementation mitigates salinity stress on *Ocimum basilicum* L. via improving water balance, ion homeostasis, and antioxidant defense system. Ecotoxicol. Environ. Saf..

[4] Sofy M.R., Elhindi K.M., Farouk S., Alotaibi M.A. (2020). Zinc and paclobutrazol mediated regulation of growth, upregulating antioxidant aptitude and plant productivity of pea plants under salinity. Plants.

[5] Yang A., Akhtar S.S., Li L., Fu Q., Li Q., Naeem M.A., He X., Zhang Z., Jacobsen S.-E. (2020). Biochar mitigates combined effects of drought and salinity stress in Quinoa. Agronomy.

[6] FAO (2017). The future of food and agriculture: Trends and challenges.

[7] Munns R., Gilliham M. (2015). Salinity tolerance of crops—What is the cost?. New Phytol..

[8] Sheng H., Zeng J., Liu Y., Wang X., Wang Y., Kang H., Fan X., Sha L., Zhang H., Zhou Y. (2020). Differential responses of two wheat varieties differing in salt tolerance to the combined stress of Mn and salinity. J. Plant Growth Regul..

[9] Ren J., Ye J., Yin L., Li G., Deng X., Wang S. (2020). Exogenous melatonin improves salt tolerance by mitigating osmotic, ion, and oxidative stresses in maize seedlings. Agronomy.

[10] Bukhat S., Manzoor H., Athar H.-U.-R., Zafar Z.U., Azeem F., Rasul S. (2019). Salicylic acid induced photosynthetic adaptability of *Raphanus sativus* to salt stress is associated with antioxidant capacity. J. Plant Growth Regul..

[11] Jiang D., Lu B., Liu L., Duan W., Chen L., Li J., Zhang K., Sun H., Zhang Y., Dong H. (2020). Exogenous melatonin improves salt stress adaptation of cotton seedlings by regulating active oxygen metabolism. Peer J.

[12] Farouk S., Al-Amri S.M. (2019). Exogenous zinc forms counteract NaCl-induced damage by regulating the antioxidant system, osmotic adjustment substances, and ions in canola (*Brassica napus* L. cv. Pactol) Plants. J. Soil Sci. Plant Nutr..

[13] Ahmad S., Cui W., Kamran M., Ahmad I., Meng X., Wu X., Su W., Javed T., El-Serehy H.A., Jia Z. (2021). Exogenous application of melatonin induces tolerance to salt stress by improving the photosynthetic efficiency and antioxidant defense system of maize seedling. J. Plant Growth Regul..

[14] Wang Y., Branicky R., Noe A., Hekimi S. (2018). Superoxide dismutases: Dual roles in controlling ROS damage and regulating ROS signaling. J. Cell Biol..

[15] Morgan J.M. (1984). Osmoregulation and water stress in higher plants. Annu. Rev. Plant Physiol..

[16] Farouk S., Al-Ghamdi A.A.M. (2021). Sodium nitroprusside application enhances drought tolerance in marjoram herb by promoting chlorophyll biosynthesis and enhancing osmotic adjustment capacity. Arab. J. Geosci..

[17] Farouk S., Omar M.M. (2020). Sweet basil growth, physiological and ultrastructural modification, and oxidative defense system under water deficit and silicon forms treatment. J. Plant Growth Regul..

[18] Hare P.D., Cress W.A., Van Staden J. (1998). Dissecting the roles of osmolyte accumulation during stress. Plant Cell Environ..

[19] Erickson C., Lehmann J., Kern D.C., Glaser B., Woods W.I. (2003). Historical ecology and future explorations. Amazonian Dark Earths: Origin, Properties, Management.

[20] Glaser B., Haumaier L., Guggenberger G., Zech W. (2001). The ’Terra Preta’ phenomenon: A model for sustainable agriculture in the humid tropics. Die Naturwissenschaften.

[21] Lehmann J., Pereira da Silva J., Steiner C., Nehls T., Zech W., Glaser B. (2003). Nutrient availability and leaching in an archaeological Anthrosol and a Ferralsol of the Central Amazon basin: Fertilizer, manure and charcoal amendments. Plant Soil.

[22] Ali E.F., Al-Yasi H.M., Kheir A.M.S., Eissa M.A. (2021). Effect of Biochar on CO_2_ sequestration and productivity of pearl millet plants grown in saline sodic soils. J. Soil Sci. Plant Nutr..

[23] Ran C., Gulaqa A., Zhu J., Wang X., Zhang S., Geng Y., Guo L., Jin F., Shao X. (2019). Benefits of biochar for improving ion contents, cell membrane permeability, leaf water status and yield of rice under saline & and ash;sodic paddy field condition. J. Plant Growth Regul..

[24] Oliveira D.M., Falcao N., Damaceno J.B.D., Guerrini I.A. (2020). Biochar yield from shell of brazil nut fruit and its effects on soil acidity and phosphorus availability in central Amazonian yellow oxisol. J. Agric. Sci..

[25] Tenic E., Ghogare R., Dhingra A. (2020). Biochar—A panacea for agriculture or just carbon?. Horticulturae.

[26] Dahlawi S., Naeem A., Rengel Z., Naidu R. (2018). Biochar application for the remediation of salt-affected soils: Challenges and opportunities. Sci. Total Environ..

[27] Ibrahim M.E.H., Ali A.Y.A., Zhou G., Elsiddig A.M.I., Zhu G., Nimir N.E.A., Ahmad I. (2020). Biochar application affects forage sorghum under salinity stress. Chil. J. Agric. Res..

[28] Zhang P., Liu L., Wang X., Wang Z., Zhang H., Chen J., Liu X., Wang Y., Li C. (2021). Beneficial effects of exogenous melatonin on overcoming salt stress in sugar beets (*Beta vulgaris* L.). Plants.

[29] Ehardeland R. (2016). Melatonin in plants; Diversity of levels and multiplicity of functions. Front. Plant Sci..

[30] Wu X., Ren J., Huang X., Zheng X., Tian Y., Shi L., Dong P., Li Z. (2021). Melatonin: Biosynthesis, content, and function in horticultural plants and potential application. Sci. Hortic..

[31] Farouk S., Al-Amri S. (2019). Ameliorative roles of melatonin and/or zeolite on chromium-induced leaf senescence in marjoram plants by activating antioxidant defense, osmolyte accumulation, and ultrastructural modification. Ind. Crop. Prod..

[32] Wei L., Zhao H., Wang B., Wu X., Lan R., Huang X., Chen B., Chen G., Jiang C., Wang J. (2021). Exogenous melatonin improves the growth of rice seedlings by regulating redox balance and ion homeostasis under salt stress. J. Plant Growth Regul..

[33] Al-Huqail A.A., Khan M.N., Ali H.M., Siddiqui M.H., Al -Huqail A., AlZuaibr F.M., Al-Muwayhi M.A., Marraiki N., Al-Humaid L. (2020). Exogenous melatonin mitigates boron toxicity in wheat. Ecotoxicol. Environ. Saf..

[34] Farouk S., Arafa S.A. (2018). Mitigation of salinity stress in canola plants by sodium nitroprusside application. Span. J. Agric. Res..

[35] Farouk S., Al-Amri S.M. (2019). Exogenous melatonin-mediated modulation of arsenic tolerance with improved accretion of secondary metabolite production, activating antioxidant capacity and improved chloroplast ultrastructure in rosemary herb. Ecotoxicol. Environ. Saf..

[36] Hu W., Yang H., Tie W., Yan Y., Ding Z., Liu Y., Wu C., Wang J., Reiter R.J., Tan D.-X. (2017). Natural Variation in Banana Varieties Highlights the Role of Melatonin in Postharvest Ripening and Quality. J. Agric. Food Chem..

[37] El-Gamal S.M.A., Serag El-Din W.M., Farouk S., Mokhtar N.A.Y.O. (2021). Integrated effects of biochar and potassium silicate on borage plant under different irrigation regimes in sandy soil. J. Hortic. Sci. Ornam. Plants.

[38] Santos C., Rodriguez E. (2012). Review on Some Emerging Endpoints of Chromium (VI) and Lead Phytotoxicity.

[39] Liu D., Kong D.D., Fu X.K., Ali B., Xu L., Zhou W.J. (2016). Influence of exogenous 5-aminolevulinic acid on chlorophyll synthesis and related gene expression in oilseed rape de-etiolated cotyledons under water-deficit stress. Photosynthetica.

[40] Kul R., Arjumend T., Ekinci M., Yildirim E., Turan M., Argin S. (2021). Biochar as an organic soil conditioner for mitigating salinity stress in tomato. Soil Sci. Plant Nutr..

[41] Chrustek A., Olszewska-Słonina D. (2020). Melatonin as a powerful antioxidant. Acta Pharm..

[42] Choi G.-H., Back K. (2019). Back suppression of melatonin 2-hydroxylase increases melatonin production leading to the enhanced abiotic stress tolerance against cadmium, senescence, salt, and tunicamycin in rice plants. Biomolecules.

[43] Hwang O.J., Back K. (2019). Melatonin deficiency confers tolerance to multiple abiotic stresses in rice via decreased brassinosteroid levels. Int. J. Mol. Sci..

[44] Weeda S., Zhang N., Zhao X., Ndip G., Guo Y., Buck G.A., Fu C., Ren S. (2014). *Arabidopsis* transcriptome analysis reveals key roles of melatonin in plant defense systems. PLoS ONE.

[45] Shi H., Zhu J.-K. (2002). Regulation of expression of the vacuolar Na^+^/H^+^ antiporter gene AtNHX1 by salt stress and abscisic acid. Plant Mol. Biol..

[46] Padan E., Venturi M., Gerchman Y., Dover N. (2001). Na^+^/H^+^ antiporters. Biochim. Biophys. Acta.

[47] Garriga M., Raddatz N., Very A.-A., Sentenac H., Rubio-Meléndez M.E., González W., Dreyer I. (2017). Cloning and functional characterization of HKT1 and AKT1 genes of *Fragaria* spp.—Relationship to plant response to salt stress. J. Plant Physiol..

[48] Zhao D., Wang R., Liu D., Wu Y., Sun J., Tao J. (2018). Melatonin and expression of tryptophan decarboxylase gene (TDC) in herbaceous peony (*Paeonia lactiflora* Pall.) flowers. Molecules.

[49] Hammer E.C., Forstreuter M., Rillig M., Kohler J. (2015). Biochar increases arbuscular mycorrhizal plant growth enhancement and ameliorates salinity stress. Appl. Soil Ecol..

[50] Abbasi H., Jamil M., Haq A., Ali S., Ahmad R., Malik Z. (2016). Parveen Salt stress manifestation on plants, mechanism of salt tolerance and potassium role in alleviating it: A review. Zemdirb.-Agric..

[51] Hafez Y., Attia K., Alamery S., Ghazy A., Al-Doss A., Ibrahim E., Rashwan E., El-Maghraby L., Awad A., Abdelaal K. (2020). Beneficial effects of biochar and chitosan on antioxidative capacity, osmolytes accumulation, and anatomical characters of water-stressed barley plants. Agronomy.

[52] Chaves M.M., Flexas J., Pinheiro C. (2009). Photosynthesis under drought and salt stress: Regulation mechanisms from whole plant to cell. Ann. Bot..

[53] Siddiqui M.H., Alamri S., Al-Khaishany M.Y., Khan M.N., Al-Amri A., Ali H.M., Alaraidh I.A., Alsahli A.A. (2019). Exogenous melatonin counteracts NaCl-induced damage by regulating the antioxidant system, proline and carbohydrates metabolism in tomato seedlings. Int. J. Mol. Sci..

[54] Trovato M., Mattioli R., Costantino P. (2008). Multiple roles of proline in plant stress tolerance and development. Rend. Lincei.

[55] Arnao M.B., Ruiz J.H. (2019). Melatonin: A new plant hormone and/or a plant master regulator?. Trends Plant Sci..

[56] Mansour M.M.F., Salama K.H.A., Hasanuzzaman M. (2020). Proline and abiotic stresses: Responses and adaptation. Plant Ecophysiology and Adaptation under Climate Change: Mechanisms and Perspectives II.

[57] Kostopoulou Z., Therios I., Roumeliotis E., Kanellis A., Molassiotis A. (2015). Melatonin combined with ascorbic acid provides salt adaptation in *Citrus aurantium* L. seedlings. Plant Physiol. Biochem..

[58] Haider I., Raza M.A.S., Iqbal R., Aslam M.U., Habib-Ur-Rahman M., Raja S., Khan M.T., Aslam M.M., Waqas M., Ahmad S. (2020). Potential effects of biochar application on mitigating the drought stress implications on wheat (*Triticum aestivum* L.) under various growth stages. J. Saudi Chem. Soc..

[59] Zhang J., Zhang Z., Shen G., Wang R., Gao L., Kong F., Zhang J. (2016). Growth performance, nutrient absorption of tobacco and soil fertility after straw biochar application. Int. J. Agric. Biol..

[60] Kongsri S., Boonprakob U., Byrne D. (2014). Assessment of morphological and physiological responses of peach rootstocks under drought and aluminum stress. Acta Hortic..

[61] Akhtar S.S., Li G., Andersen M.N., Liu F. (2014). Biochar enhances yield and quality of tomato under reduced irrigation. Agric. Water Manag..

[62] Yeboah S., Zhang R., Cai L., Li L., Xie J., Luo Z., Wu J., Antille D.L. (2017). Soil water content and photosynthetic capacity of spring wheat as affected by soil application of nitrogen-enriched biochar in a semiarid environment. Photosynthetica.

[63] Kammann C.I., Linsel S., Gobling J.W., Koyro H.W. (2011). Influence of biochar on drought tolerance of *Chenopodium quinoa* Willd and on soil-plant relations. Plant Soil.

[64] Sanders G.J., Arndt S.K., Aroca R. (2012). Osmotic adjustment under drought conditions. Plant Responses to Drought Stress.

[65] Munns R., Passioura J.B., Colmer T.D., Byrt C.S. (2019). Osmotic adjustment and energy limitations to plant growth in saline soil. New Phytol..

[66] Shahid M., Shamshad S., Rafiq M., Khalid S., Bibi I., Niazi N.K., Dumat C., Rashid M.I. (2017). Chromium speciation, bioavailability, uptake, toxicity and detoxification in soil-plant system: A review. Chemosphere.

[67] Kataria S., Baghel L., Jain M., Guruprasad K.N. (2019). Magnetopriming regulates antioxidant defense system in soybean against salt stress. Biocatal. Agric. Biotechnol..

[68] Campos M.L.D.O., de Hsie B.S., Granja J.A.D.A., Correia R.M., de Almeida-Cortez J.S., Pompelli M.F. (2012). Photosynthesis and antioxidant activity in *Jatropha curcas* L. under salt stress. Braz. J. Plant Physiol..

[69] Wang L.Y., Liu J.L., Wang W.X., Sun Y. (2016). Exogenous melatonin improves growth and photosynthetic capacity of cucumber under salinity-induced stress. Photosynthetica.

[70] Torabian S., Farhangi-Abriz S., Rathjen J. (2018). Biochar and lignite affect H^+^-ATPase and H^+^-PPase activities in root tonoplast and nutrient contents of mung bean under salt stress. Plant Physiol. Biochem..

[71] Gill R.A., Zang L., Ali B., Farooq M.A., Cui P., Yang S., Ali S., Zhou W. (2015). Chromium-induced physio-chemical and ultrastructural changes in four cultivars of *Brassica napus* L. Chemosphere.

[72] Pandey V., Dixit V., Shyam R. (2009). Chromium effect on ROS generation and detoxification in pea (*Pisum sativum*) leaf chloroplasts. Protoplasma.

[73] Wang J., Chen J., Sharma A., Tao S., Zheng B., Landi M., Yuan H., Yan D. (2019). Melatonin stimulates activities and expression Level of antioxidant enzymes and preserves functionality of photosynthetic apparatus in hickory plants (*Carya cathayensis* Sarg.) under PEG-promoted drought. Agronomy.

[74] Kulbat K. (2016). The role of phenolic compounds in plant resistance. Biotechnol. Food Sci..

[75] Foyer C.H. (2018). Reactive oxygen species, oxidative signaling and the regulation of photosynthesis. Environ. Exp. Bot..

[76] Michalak A. (2006). Phenolic compounds and their antioxidant activity in plants growing under heavy metal stress. Pol. J. Environ. Stud..

[77] Liang W., Ma X., Wan P., Liu L. (2018). Plant salt-tolerance mechanism: A review. Biochem. Biophys. Res. Commun..

[78] Kiani R., Arzani A., Maibody S.A.M.M. (2021). Polyphenols, flavonoids, and antioxidant activity involved in salt tolerance in wheat, *Aegilops cylindrica* and their amphidiploids. Front. Plant Sci..

[79] Leopoldini M., Russo N., Chiodo A.S., Toscano M. (2006). Iron chelation by the powerful antioxidant flavonoid quercetin. J. Agric. Food Chem..

[80] Khatun S., Flowers T.J. (1995). Effects of salinity on seed set in rice. Plant, Cell Environ..

[81] Qi Z.-Y., Wang K.-X., Yan M.-Y., Kanwar M.K., Li D.-Y., Wijaya L., Alyemeni M.N., Ahmad P., Zhou J. (2018). Melatonin alleviates high temperature-induced pollen abortion in *Solanum lycopersicum*. Molecules.

[82] Hu W., Cao Y., Loka D.A., Harris-Shultz K.R., Reiter R.J., Ali S., Liu Y., Zhou Z. (2020). Exogenous melatonin improves cotton (*Gossypium hirsutum* L.) pollen fertility under drought by regulating carbohydrate metabolism in male tissues. Plant Physiol. Biochem..

[83] Huang M., Yang L., Qin H., Jiang L., Zou Y. (2014). Fertilizer nitrogen uptake by rice increased by biochar application. Biol. Fertil. Soils.

[84] El-Naggar A., Lee S.S., Rinklebe J., Farooq M., Song H., Sarmah A.K., Zimmerman A.R., Ahmad M., Shaheen S.M., Ok Y.S. (2019). Biochar application to low fertility soils: A review of current status, and future prospects. Geoderma.

[85] Zhang W.-M., Meng J., Wang J.-Y., Fan S.-X., Chen W.-F. (2013). Effect of biochar on root morphological and physiological characteristics and yield in rice. Acta Agron. Sin..

[86] Gollan T., Schurr U., Schulze E.-D. (1992). Stomatal response to drying soil in relation to changes in the xylem sap composition of *Helianthus annuus*. I. The concentration of cations, anions, amino acids in, and pH of, the xylem sap. Plant Cell Environ..

[87] Wilkinson S., Davies W.J. (1997). Xylem sap pH increase: A drought signal received at the apoplastic face of the guard cell that involves the suppression of saturable abscisic acid uptake by the epidermal symplast. Plant Physiol..

[88] El-Mallah M.H., Turui T., El-Shami S. (1994). Detailed studies on seed oil of *Salicornia* SOS-7 cultivated at the Egyptian border of Red Sea. Grasas y Aceites.

[89] Eganathan P., Subramanian H.M.S.R., Latha R., Rao C.S. (2006). Oil analysis in seeds of *Salicornia brachiata*. Ind. Crop. Prod..

[90] Weber D., Ansari R., Gul B., Khan M.A. (2007). Potential of halophytes as source of edible oil. J. Arid Environ..

[91] Motsara M.R., Roy R.N. (2008). Guide to Laboratory Establishment for Plant Nutrient Analysis.

[92] Koller H.R.C. (1972). Leaf area-leaf weight relationship in soybean canopy. Crop Sci..

[93] Lichtenthaler H.K., Wellburn A.R. (1983). Determinations of total carotenoids and chlorophylls a and b of leaf extracts in different solvents. Biochem. Soc. Trans..

[94] Fatma M., Asgher M., Masood A., Khan N.A. (2014). Excess sulfur supplementation improves photosynthesis and growth in mustard under salt stress through increased production of glutathione. Environ. Exp. Bot..

[95] Malik R.N., Husain S.Z., Nazir I. (2010). Heavy metal contamination and accumulation in soil and wild plant species from industrial area of Islamabad, Pakistan. Pak. J. Bot..

[96] Kojić D., Pajević S., Jovanović-Galović A., Purać J., Pamer E., Škondrić S., Milovac S., Popović Ž., Grubor-Lajšić G. (2012). Efficacy of natural aluminosilicates in moderating drought effects on the morphological and physiological parameters of maize plants (*Zea mays* L.). J. Soil Sci. Nutr..

[97] Sadasivam S., Manickam A. (2008). Biochemical Methods.

[98] Kaya C., Sonmez O., Aydemir S., Ashraf M., Dikilitas M. (2013). Exogenous application of mannitol and thiourea regulates plant growth and oxidative stress responses in salt-stressed maize (*Zea mays* L.). J. Plant Interact..

[99] Baque M.A., Karim M.A., Hamida A. (2002). Role of potassium on water relation behavior of *Triticum aestivum* L. under water stress conditions. Prog. Agric..

[100] Taiz L., Zeiger E. (2015). Plant Physiology and Development.

[101] Nobel P.S. (1991). Physiochemical and Environmental Plant Physiology.

[102] Blum A. (1989). Osmotic adjustment and growth of barley genotypes under drought stress. Crop Sci..

[103] Aftab T., Khan M.M.A., da Silva J.A.T., Idrees M., Naeem M. (2011). Role of salicylic acid in promoting salt stress tolerance and enhanced artemisinin production in *Artemisia annua* L. J. Plant Growth Regul..

[104] Djanaguiraman M., Prasad P., Seppanen M. (2010). Selenium protects sorghum leaves from oxidative damage under high temperature stress by enhancing antioxidant defense system. Plant Physiol. Biochem..

[105] Levine R.L., Williams J.A., Stadtman E.P., Shacter E. (1994). Carbonyl assays for determination of oxidatively modified proteins. Methods Enzymol..

[106] Chrysargyris A., Xylia P., Botsaris G., Tzortzakis N. (2017). Antioxidant and antibacterial activities, mineral and essential oil composition of spearmint (*Mentha spicata* L.) affected by the potassium levels. Ind. Crop. Prod..

[107] Alp S., Ercisli S., Jurikova T., Cakir O., Gozlekci S. (2016). Bioactive content of rose hips of different wildly grown *Rosa dumalis* genotypes. Not. Bot. Horti Agrobot. Cluj-Napoca.

[108] AOAC (2000). Official Methods of Analysis of the Association of Official Analytical Chemists.

